# Extracellular matrix reorganization during endometrial decidualization

**DOI:** 10.1007/s00418-025-02411-3

**Published:** 2025-08-28

**Authors:** Mona Gebril, Sparhawk Mulder, Rimi Das, Shanmugasundaram Nallasamy

**Affiliations:** 1 Department of Obstetrics, Gynecology, and Reproductive Sciences, University of Vermont College of Medicine, Burlington, VT USA; 2 Division of Reproductive Sciences, Department of Obstetrics, Gynecology and Reproductive Sciences, University of Vermont College of Medicine, 89 Beaumont Avenue, Burlington, VT 05405 USA

**Keywords:** Mouse endometrium, Decidualization, Extracellular matrix, Collagen, Elastic fibers, Lysyl oxidases

## Abstract

Extracellular matrix reorganization, a concurrent process of endometrial decidualization, has garnered widespread recognition. However, our understanding of this process remains limited. In this study, we aimed to investigate the expression, spatial distribution, and reorganization of fibrillar collagens, elastin, and lysyl oxidases within the decidua. Using second harmonic generation imaging, we successfully recorded fibrillar collagen reorganization between preimplantation and decidualized endometrium. Upon embryo implantation, the fibrillar collagens align themselves parallel to the direction of embryo invasion. Furthermore, we employed confocal imaging analysis to reveal distinct expression and spatial distribution patterns of elastin and lysyl oxidase-like enzymes. Elastin expression begins to manifest surrounding the implanting embryo, extends into the decidua, and exhibits a high concentration in the mesometrial region after gestational day 8. All lysyl oxidase-like enzymes are localized within the decidua, although they exhibit varying expression patterns. To gain further insights, we utilized an in vitro stromal cell decidualization model and provided compelling evidence that stromal cells serve as the primary source of the extracellular matrix components during endometrial decidualization. Additionally, we demonstrated that the genes encoding factors involved in the synthesis, processing, and assembly of fibrillar collagen and elastic fibers exhibit differential expression patterns during in vitro decidualization. Genes such as asporin, decorin, thrombospondin 2, fibulin 2, fibulin 5, and lysyl oxidase show significant induction during in vitro decidualization. In summary, our comprehensive analysis provides a detailed evaluation of the expression, spatial distribution, and reorganization of fibrillar collagens, elastin, and lysyl oxidases during the process of endometrial decidualization.

**Supplementary Information:**

The online version contains supplementary material available at 10.1007/s00418-025-02411-3.

## Introduction

Embryo implantation, a pivotal step in establishing a successful pregnancy, is a multifaceted process characterized by a harmonious interplay of cellular and molecular events (Ochoa-Bernal and Fazleabas 2020; Cha et al. 2012; Muter et al. 2023). During implantation, the embryo adheres to the uterine epithelium and invades into the uterine stromal compartment (Akaeda et al. 2024). This process initiates extensive proliferation and differentiation of stromal cells, leading to the formation of a transient structure called the “decidua” (Ramathal et al. 2010; Vinketova et al. 2016). The decidua has remarkable capabilities to receive, host, and nurture the embryo until it establishes a more sophisticated communication channel through placentation (Gellersen and Brosens 2014). Throughout this period, the decidua must maintain its functional homeostasis and structural integrity while simultaneously regulating the invasion of trophoblast cells. The decidua achieves this in part through the synthesis and reorganization of its extracellular matrix (ECM).

The tissue-specific organization of the ECM is meticulously orchestrated during embryonic development. In adulthood, the ECM undergoes limited reorganization, with the exception of reproductive tissues (Bonnans et al. 2014; Nallasamy et al. 2017; Liu et al. 2004). ECM reorganization, the continuous and regulated process of degrading and synthesizing new components, is pivotal for tissue development, repair, and homeostasis (Lu et al. 2011). This reorganization is one of the fundamental events underlying decidualization triggered by embryo invasion (Rossi et al. 2025; Favaro et al. 2014; O’Connor et al. 2020; Shi et al. 2020). Aberrant alterations in the structure and composition of the ECM in the endometrium have been associated with pathological conditions such as recurrent miscarriage, endometriosis, adenomyosis, and endometrial aging (Pathare et al. 2023; Cheong et al. 2019; Rossi et al. 2025; Kim et al. 2019; Ruiz et al. 2015; Herndon et al. 2016). Understanding the physiological reorganization process is essential for effectively managing disorders caused by abnormal ECM reorganization.

The ECM is a sophisticated and dynamic network of proteins and polysaccharides that offers structural and biochemical support to surrounding cells (Mouw et al. 2014; Cox 2021; Naba 2024). It plays a crucial role in maintaining tissue integrity, facilitating cellular communication, and regulating various physiological processes (Chaudhuri et al. 2020; Humphrey et al. 2014). The ECM comprises a diverse array of macromolecules, including collagens, elastin, glycoproteins, proteoglycans, and glycosaminoglycans (Mouw et al. 2014; Theocharis et al. 2016). Fibrillar collagens, the most abundant ECM proteins, provide tensile strength and structural support (Ricard-Blum 2011). Elastin offers tissues resilience, enabling them to return to their original shape following stretching or contraction (Wagenseil and Mecham 2009). The lysyl oxidase family of enzymes play a crucial role in determining the strength of collagen and elastic fibers by catalyzing covalent cross-linking reactions (Barker et al. 2012). Some of these factors are reported to be expressed in mouse decidua (Spiess et al. 2007; Li et al. 2017). However, the precise organization of multiple ECM components within the decidua, along with their spatial distribution, remains less well understood.

The study offers a detailed examination of the expression, spatial distribution, and reorganization of fibrillar collagens, elastic fibers, and lysyl oxidases within the decidua during embryo implantation. Moreover, it presents compelling experimental evidence suggesting that the endometrial stromal cells serve as the primary source of these proteins during decidualization.

## Methods

### Mice and tissue collection

C57BL/6J129Sv mice were used in this study. To collect timed-pregnant tissues, breeding pairs were mated in the morning, and vaginal plugs were checked in the afternoon. The presence of a vaginal plug was designated as day 0 of pregnancy. Pregnant uteri were collected from gestational days 3 to 9. Implantation sites were harvested and prepared as frozen tissue blocks by immersing them in OCT (Tissue-Tek, Thermo Fisher Scientific, Inc., Waltham, MA, USA) and solidifying them with liquid nitrogen vapor. The blocks were stored at −80 °C until processing. All animal experiments were conducted in accordance with the National Institutes of Health Guide for the Care and Use of Laboratory Animals humane animal care standards, and were approved by the Institutional Animal Care and Use Committee of the University of Vermont.

### Second harmonic generation imaging

Frozen uterine cross sections of 50 μm thickness were utilized in this experiment. The thawed sections were subsequently covered with 0.1 M phosphate-buffered saline (PBS) to preserve the hydration of the tissue sections during imaging. A Zeiss LSM7 inverted microscope (Carl Zeiss AG, Oberkochen, Germany) equipped with an Achroplan 20x/0.8 objective lens was employed to acquire images of the slides. A Chameleon XR pulsed Ti: sapphire laser (Coherent, Santa Clara, CA, USA) was adjusted to emit a wavelength of 900 nm onto the tissue, resulting in a signal detected at 450 nm. The acquired images were subsequently analyzed using ImageJ software (National Institutes of Health).

### Confocal microscopic imaging

Frozen uterine cross sections (5 µm thick) were fixed in acetone for 10 min and subsequently blocked with 10% normal goat serum (Life Technologies, Carlsbad, CA, USA) for 30 min at room temperature. Primary antibodies—COL1A1 (Cell Signaling Technology, Danvers, MA, USA; 72026, RRID:AB_2904565, 1:200), collagen 3 (Proteintech, Rosemont, IL, USA; 22734-1-AP, RRID:AB_2879158, 1:500), elastin (Elastin Products Company, Owensville, MO, USA; PR385, RRID:AB_3099530, 1:250), LOXL1 (Thermo Fisher Scientific, Inc., Waltham, MA, USA; PA5-87701, RRID:AB_2804357, 1:500), LOXL2 (Abcam, Waltham, MA, USA; Ab96233, RRID:AB_10677617, 1:100), LOXL3 (Thermo Fisher Scientific, Inc., Waltham, MA, USA; PA5-48462, RRID:AB_2633919, 1:500), LOXL4 (Thermo Fisher Scientific, Inc., Waltham, MA, USA; PA5-115520, RRID:AB_2900156, 1:100), and smooth muscle actin (Santa Cruz Biotechnology, Inc, Dallas TX, USA; sc-32251, RRID:AB_262054, 1:1000)—were diluted in blocking solution, added to the tissue sections. The slides were then incubated overnight at 4 °C. The following day, slides were washed with PBS and then incubated with secondary antibodies—anti-rabbit IgG Alexa Fluor 555 (Thermo Fisher Scientific Inc., Waltham, MA, USA; A32732, RRID: AB_2633281, 1:500) and anti-mouse IgG Alexa Fluor 488 (Thermo Fisher Scientific, Inc., Waltham, MA, USA; A11029, RRID: AB_2534088, 1:500)—in blocking solution for 30 min at room temperature. Slides were washed with PBS and mounted with ProLong Gold Antifade Mountant with DNA Stain DAPI (Thermo Fisher Scientific, Inc., Waltham, MA, USA). Images were acquired using a Nikon A1R confocal microscope (Nikon Instruments, Melville, NY, USA) equipped with a galvanometer scanner and illumination point scan at a rate of eight frames per second for a 1024 × 1024-pixel field. Both 4× and 10× objective lenses were used for imaging. Nikon NIS-Elements software (Nikon Instruments, Melville, NY) was used for image acquisition. The acquired images were further analyzed using ImageJ (National Institutes of Health).

### Mouse endometrial stromal cell culture

On gestational day 3, the pregnant uteri were collected, sectioned longitudinally, and subsequently cut into 3–5 mm pieces. These tissues were subsequently digested with a mixture of 6 g/L dispase (07923, STEMCELL Technologies, Cambridge, MA, USA), 25 g/L pancreatin, and antibiotic–antimycotic solution ( Thermo Fisher Scientific, Inc., Waltham, MA, USA) in Hank's Balanced Salt Solution (HBSS) for 1 h at room temperature, followed by 15 min at 37 °C. After aspirating the enzyme mixture, the tissues were then digested with 250 µg/mL Liberase (Sigma-Aldrich, Saint Louis, MO, USA) in HBSS (Thermo Fisher Scientific, Inc., Waltham, MA, USA) for 45 min at 37 °C. The enzyme action was terminated by adding 10% fetal bovine serum (FBS; Thermo Fisher Scientific, Inc., Waltham, MA, USA). The mixture was filtered through a 70 µm strainer and subsequently centrifuged. The resulting pellet was diluted in DMEM-F12 (Thermo Fisher Scientific, Inc., Waltham, MA, USA) containing 2% FBS, 1% antibiotic and antimycotic solution, 1 µM progesterone (Sigma-Aldrich, Saint Louis, MO, USA), and 10 nM 17β-estradiol (Sigma-Aldrich, Saint Louis, MO, USA). Cells were counted using a hemocytometer and were plated in six-well plates at a concentration of 1 × 10^6^ cells/well. After 2–3 h, the culture medium was replaced, and thereafter the culture medium was replaced daily. Cell lysate and conditioned media were collected at 24, 48, 72, and 96 h for gene and protein expression analysis.

### Quantitative polymerase chain reaction (qPCR)

Total RNA was extracted from stromal cell lysates using the RNeasy Mini Kit (74104, Qiagen, Germantown, MD, USA), following the manufacturer’s protocol. Complementary DNA (cDNA) was synthesized using the iScript Reverse Transcription Supermix (Bio-Rad Laboratories, Hercules, CA, USA). Quantitative PCR was performed using SYBR Green (Thermo Fisher Scientific, Inc., Waltham, MA, USA) and primers specifically designed for the genes of interest. Gene expression was quantified using the 2^–ΔΔCt^ method, with target gene expression normalized to the housekeeping gene *Rplp0*. The primers used in this study are listed in Table 1.

**Table 1:** The list of primers used in this study

Oligo name	Forward	Reverse
*Aspn*	TCCTCTGACAAGGTTGGACT	TTCAGTGCTGTGTGGGAAGG
*Bgn*	TGTCCCTCCCCAGCAATCTA	CTCTACTCCCTCTTTGTGCCC
*Bmp1*	GTTCCCAAGTATGAGGTGAATGG	CGGGCCTGAGCGATGTC
*Col1a1*	CAACCTGGACGCCATCAAG	CAGACGGCTGAGTAGGGAACA
*Col3a1*	TGTCCTTTGCGATGACATAATCTG	TGTGGGCATATTGCACAACAT
*Dcn*	AACTGTGCTATGGAGTAGAAGCA	ATCTCATGTATTTTCACGACCTTTT
*Efemp1*	CGCCAGTTCAGACCTACCAG	TATCCATCGGTGCATTGCGT
*Eln*	CAGTACTGTAACCCCGTTCTTCCT	GATCTATCACAGCGAAACACAAAGAG
*Emilin*	CCACTGTTCCCGAAGTATCATG	GTCTTGTAAGCCACCCGATAGC
*Fbln1*	AGCAGCACTCCACCCAAAAG	ACCAAAATGCCAATCCTCATG
*Fbln2*	TCGTCACACGCAGACTCAATG	CCCGCGGCTCCAGAAC
*Fbln4*	GAGAGCAGCCTTCATCCATTG	ACACGTCAGCAGGCACACTTC
*Fbln5*	GATGCAAGCAACAACCCGATA	CCCTCGTTGCCAGATTTGAT
*Fbn1*	GCCAGAACCTTCACATCATGGT	GAGAAAGTGGTTGGTTGAACTAGGA
*Fbn2*	AGGAGCACAATGAGGACGAC	CCAAACACTTCTGAAGGCCG
*Fmod*	GGGCAACAGGATCAATGAGTTC	GATCTCGATGAGGTTGGCGA
*Gja1*	TGGACAAGGTCCAAGCCTACTC	TCCCCAGGAGCAGGATTCT
*Lox*	ACTGCTACGATTTCCGCAAAG	CGAGGGCGGCTTGGTAA
*Loxl1*	CAACGTGGTGAGATGCAACAT	GGACGATTTTGCAGTTTGTTGTAG
*Loxl2*	GCTGGCCCAATGTTTTCATC	AAGATTATAAGGTGGCTCCAGAGGTA
*Loxl3*	GGGCATTACCACAGTATGGACAT	GCCACCTTGGTGCCATTG
*Loxl4*	GTGAACCCCACAAACGATGTT	GCGGCACCGTATCATGTTATT
*Pcolce*	CCTCCAGAGAGCTTCGTGGTT	GACACTTCCTCTTGCTTAGGTTATTGA
*Pcolce2*	TGGCTTCATGGCAACGTACT	AATCTCGGTCTGGCCAGTTG
*Prl8a2*	AAACCCACCAGCTCATGGAC	GGAGTGATCCATGCACCCAT
*Rplpo*	CACTGGTCTAGGACCCGAGAAG	GGTGCCTCTGGAGATTTTCG
*Thbs1*	GCAAAGACGTCGATGAGTGC	CGGTTTGCACACCTGTTTGT
*Thbs2*	AGAGAGAGCCAGTCCGATGT	TCTTCACGTGACCTGGTGTG
*Tll1*	CGTGCAAAGCTGCTGTGTTT	ATGGTCTCCAAAGCCACCTG

### Western blot

Endometrial stromal cells were lysed using radioimmunoprecipitation assay (RIPA) buffer containing 1% protease and phosphatase inhibitors (Thermo Fisher Scientific Inc., Waltham, MA, USA) and stored at −80 °C until processing. Protein concentration was determined using a bicinchoninic acid (BCA) protein assay (Thermo Fisher Scientific, Inc., Waltham, MA, USA). Samples (20 µg) were boiled at 95 °C for 10 min in Laemmli Sample Buffer (Bio-Rad Laboratories, Hercules, CA, USA) with β-mercaptoethanol (Sigma-Aldrich, Saint Louis, MO, USA). The samples and a protein standard (Precision Plus Protein Kaleidoscope, Bio-Rad Laboratories, Hercules, CA, USA) were loaded into a 10% Bis/Tris–HCl sodium dodecyl sulfate–polyacrylamide gel electrophoresis (SDS-PAGE) gel and separated at 50 V for 10 min, followed by 100 V for 1 h. Proteins were then transferred onto a nitrocellulose membrane (Bio-Rad Laboratories, Hercules, CA, USA) at 100 V for 1 h at 4 °C. Membranes were blocked with 3% blotting-grade nonfat dry milk in TBST (Bio-Rad Laboratories, Hercules, CA, USA) for 1 h at room temperature. Primary antibodies used were as follows: COL1A1 (Cell Signaling Technology, Danvers, MA, USA; 72026, RRID:AB_2904565, 1:1000), collagen 3 (Proteintech, Rosemont, IL, USA; 22734-1-AP, RRID:AB_2879158, 1:1000), LOX (Abcam, Waltham, MA, USA; Ab174316, RRID:AB_2630343, 1:1000), LOXL1 (Thermo Fisher Scientific, Inc., Waltham, MA, USA; PA5-87701, RRID:AB_2804357, 1:500), LOXL2 (Novus Biologicals LLC, Minneapolis, MN, USA, NBP1-32954, RRID:AB_10677617, 1:500), LOXL3 (Thermo Fisher Scientific, Inc., Waltham, MA, USA; PA5-48462, RRID:AB_2633919, 1:1000), LOXL4 (Thermo Fisher Scientific, Inc., Waltham, MA, USA; PA5-115520, RRID:AB_2900156, 1:500), and GAPDH (Cell Signaling Technology, Danvers, MA, USA; 97166, RRID:AB_2756824, 1:500). All primary antibodies were incubated overnight at 4 °C in blocking solution. Secondary antibodies labeled with horseradish peroxidase (HRP) (goat anti-rabbit IgG (H/L): HRP, Cell Signaling Technology, Danvers, MA, USA; 7074S, 1:1000; goat anti-mouse IgG (H/L): HRP, Cell Signaling Technology, Danvers, MA, USA; 7076S, 1:1000) were added for 1 h at room temperature. Imaging was conducted using Amersham ECL Western Blotting Detection Reagents and ImageQuant 800 Western blot imaging systems (Cytiva Life Sciences, Marlborough, MA, USA).

### Statistical analysis

Data were collected and analyzed using Prism software (GraphPad Software, Boston, MA, USA). A one-way analysis of variance (ANOVA) followed by Dunnett’s multiple comparisons test was used to compare multiple groups. Values are presented as the mean ± standard error of the mean (SEM), with statistical significance determined at *p* < 0.05.

## Results

### Fibrillar collagen reorganization within the mouse decidua during embryo implantation

**Fig. 1:**
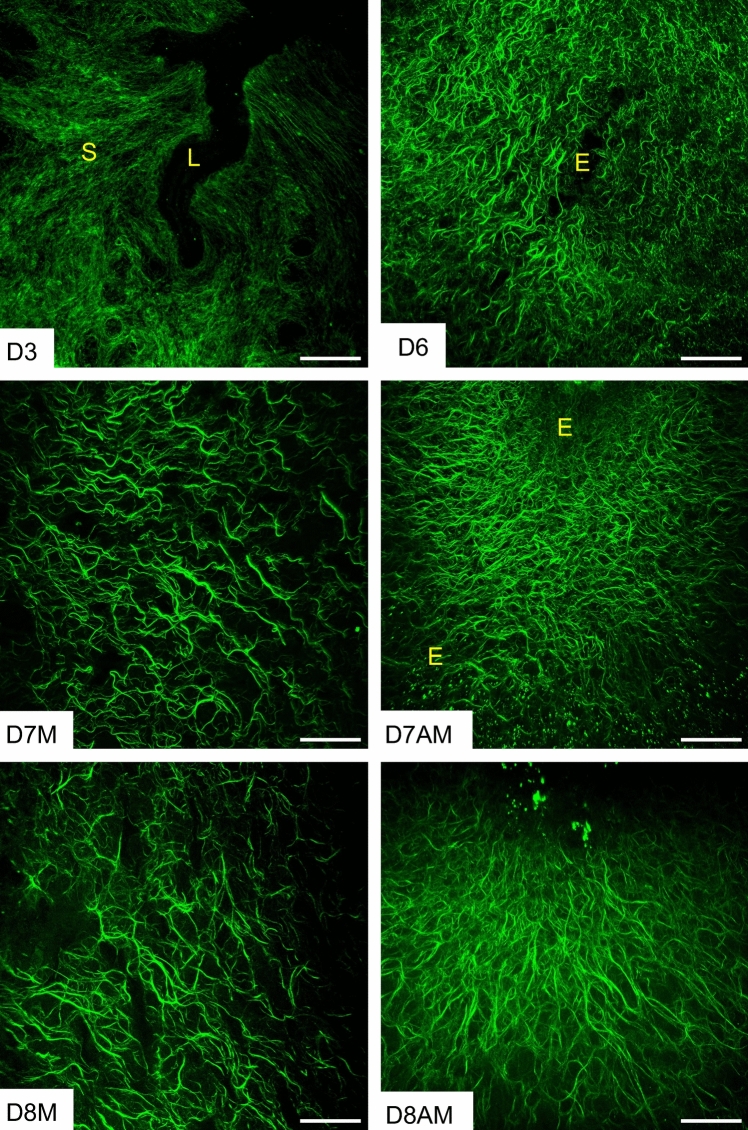
Structural reorganization of fibrillar collagen in the preimplantation and decidualized mouse uterus. Second harmonic generation imaging of collagen fibers on gestational day 3 (D3), day 6 (D6), day 7 [mesometrial region (D7M)/anti-mesometrial region (D7AM)], and day 8 [mesometrial region (D8M)/anti-mesometrial region (D8AM)] in uteri (*n* = 3/gestational time points). The Z-stack images taken at 5 μm intervals deeper into 50 μm thick frozen sections were projected using ImageJ. Autofluorescence signals elicited by collagen fibers are pseudo-colored in green. The imaging settings were adjusted for each individual image to optimize the visualization of morphology. *L* uterine lumen, *S* stroma, and *E* embryo. Scale bar: 100 μm

Second harmonic generation (SHG) imaging is a powerful tool that spatially resolves the fibrillar collagen morphology in tissues (Nallasamy et al. 2017; Campagnola 2011). Therefore, we utilized SHG imaging to examine the collagen fiber morphology during endometrial decidualization. To visualize the collagen fiber network deeper within the tissues, we acquired Z-stack images at 5 µm intervals through 50 µm-thick uterine cross sections. On gestational day 3 of the preimplantation uterus, the collagen fibers are discernible as thin strands that are evenly distributed throughout the stromal compartment. In contrast, we discovered a remarkably dense network of fibrillar collagen surrounding the invading embryo within the decidua from gestational days 6 to 8 (Fig. 1). The collagen fibers oriented themselves in parallel to each other but also parallel to the invading embryo, implying that these fibers potentially determine the direction of embryo invasion from the mesometrial to the anti-mesometrial end within the decidua on gestational days 7 and 8 (Fig. 1). SHG imaging revealed distinct sources and characteristics of fibrillar collagen between the mesometrial and anti-mesometrial regions. In the anti-mesometrial region, the fibrillar collagens exhibited a filament-like structure due to signals from the decidual ECM. Conversely, in the mesometrial region, they appeared tubular with branching due to signals primarily elicited by the vascular ECM on gestational days 7 and 8 (Fig. 1). These findings elucidate that fibrillar collagen reorganization constitutes a distinct underlying process during decidualization.

### Localization and spatial distribution of fibrillar collagens 1 and 3 in the mouse decidua

The visualization of the reorganization of fibrillar collagen by SHG led to the subsequent step of identifying the localization and spatial distribution of the primary fibrillar collagen subtypes in the decidua. To comprehend the localization and spatial distribution of the predominant fibrillar collagens, such as collagen 1 and 3, over the course of embryo implantation and decidualization, confocal imaging was employed. Consistent with SHG imaging, collagen 1 was abundantly and evenly distributed throughout the decidua. Furthermore, the collagen 1 staining exhibited a reorganization pattern surrounding the embryo (Supplementary Fig. 1). In contrast, collagen 3 was distributed evenly in the endometrium of a gestational day 3 pregnancy (Supplementary Fig. 2). Notably, from gestational day 5 onwards, the spatial distribution of collagen 3 was drastically reduced within the decidua. The spatial distribution gradually faded away from the decidua but persisted surrounding the embryo and in the deep stromal compartment (Supplementary Fig. 2). These results demonstrate that fibrillar collagen 1 is the predominant type of collagen present within the decidua and undergoes distinct reorganization during decidualization.

### Synthesis of fibrillar collagen by endometrial stromal cells in vitro

**Fig. 2:**
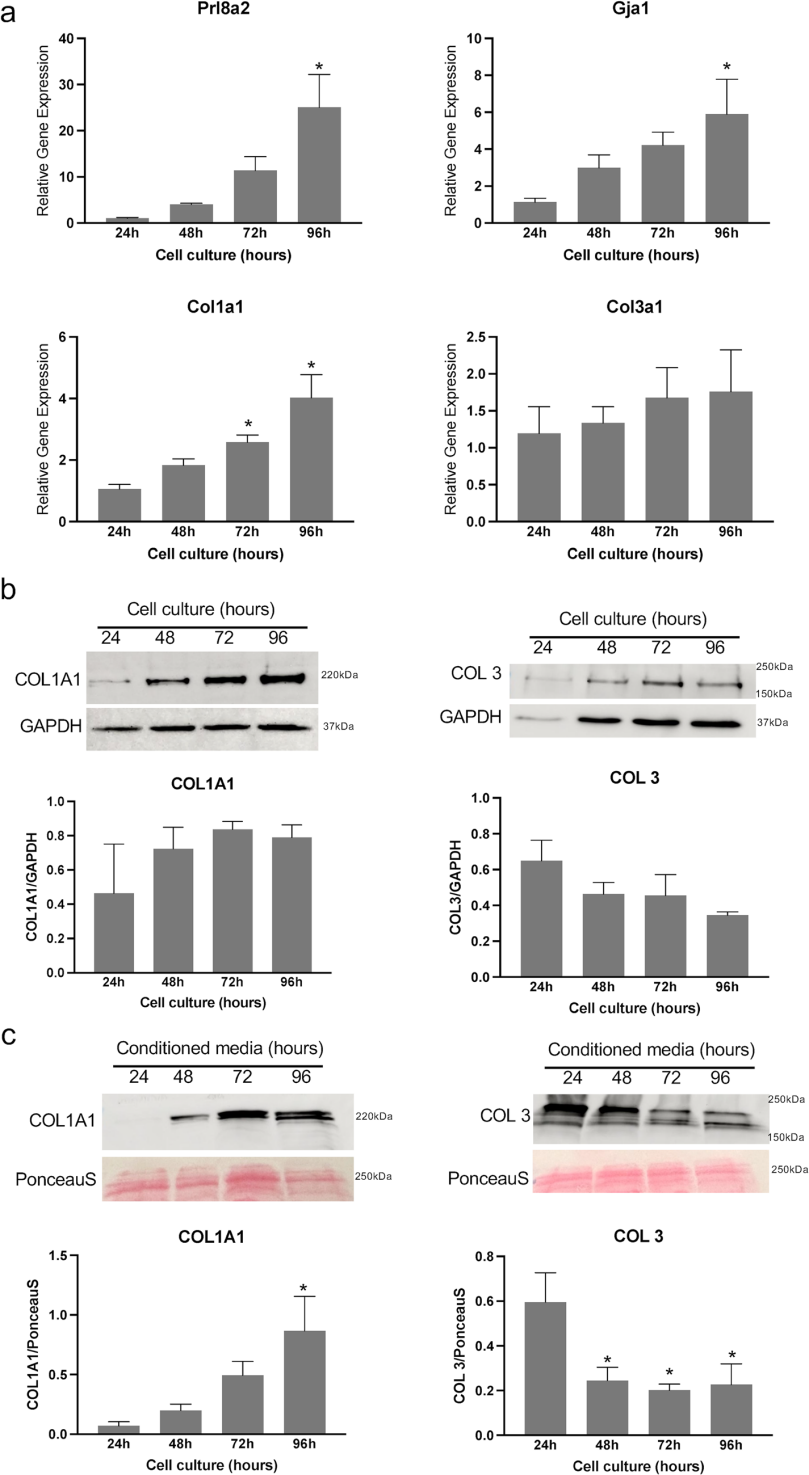
Gene expression and protein levels of fibrillar collagen 1 and 3 in mouse endometrial stromal cells decidualized in vitro. **a** Gene expression of Prl8a2 and Gja1 and genes encoding collagen subunits, Col1a1 and Col3a1, in the mouse endometrial stromal cells decidualized in vitro. Mouse endometrial stromal cells isolated from gestational day 3 uteri were cultured in vitro. The cell lysates were collected at 24, 48, 72, and 96 h to extract total RNA for gene expression analysis. The gene expression was normalized to Rplp0 and compared with 24-h samples (*n* = 6/group, *p < 0.05). **b** Western blot analysis of COL1A1 and collagen 3 protein levels in cell lysates collected at 24, 48, 72, and 96 h. GAPDH was used as a loading control. These are representative images from three independent replicates. Quantification of protein levels of COL1A1 and collagen 3 is shown in histogram. **c** Western blot analysis of COL1A1 and collagen 3 protein levels in the conditioned media collected at 24, 48, 72, and 96 h after culture. These are representative images from four independent replicates. Note: The culture medium was replaced every day. PonceauS stain was used to assess transfer efficiency and to visualize total proteins. Quantification of protein levels of COL1A1 and collagen 3 is shown in histogram (*n* = 4/group, *p < 0.05)

Our understanding of fibrillar collagen reorganization in the decidua prompted us to identify the role of decidual cells in collagen synthesis. We hypothesize that the endometrial stromal cells synthesize fibrillar collagens within the decidua. To test this hypothesis, we decidualized endometrial stromal cells in vitro and analyzed their gene and protein expression. We collected and purified endometrial stromal cells from gestational day 3 uteri and cultured them in vitro. At 24, 48, 72, and 96 h after culture, we collected cell lysates to analyze gene and protein expression. Additionally, we collected conditioned media to estimate the levels of collagen 1 and 3, as these are secreted factors. To validate our decidualization system, we analyzed the gene expression of well-known markers of decidualization, such as Prl8a2 and Gja1. We observed a gradual increase in the gene expression of Prl8a2 and Gja1 from 24 to 96 h after culture (Fig. 2a).

The gene expression of Col1a1 gradually increased and reached a significant level at 72 h, with a maximum at 96 h, compared to 24 h. On the other hand, the gene expression of Col3a1 remained relatively constant throughout the time points tested (Fig. 2a). We were able to identify the COL1A1 and collagen 3 proteins in the cell lysates, but their levels did not show significant differences (Fig. 2b). In contrast, the protein levels of COL1A1 exhibited a steady increase in the conditioned media collected and reached a statistically significant level at 96 h. Conversely, the levels of collagen 3 were significantly reduced at all time points compared to 24 h (Fig. 2c). These results correlate with localization studies described above for collagen 1 and 3 (Supplementary Figs. 1 and 2). These findings collectively demonstrate that endometrial decidual cells are capable of synthesizing fibrillar collagen during decidualization.

### Expression of genes encoding factors involved in collagen synthesis, processing, and assembly during in vitro decidualization

**Fig. 3:**
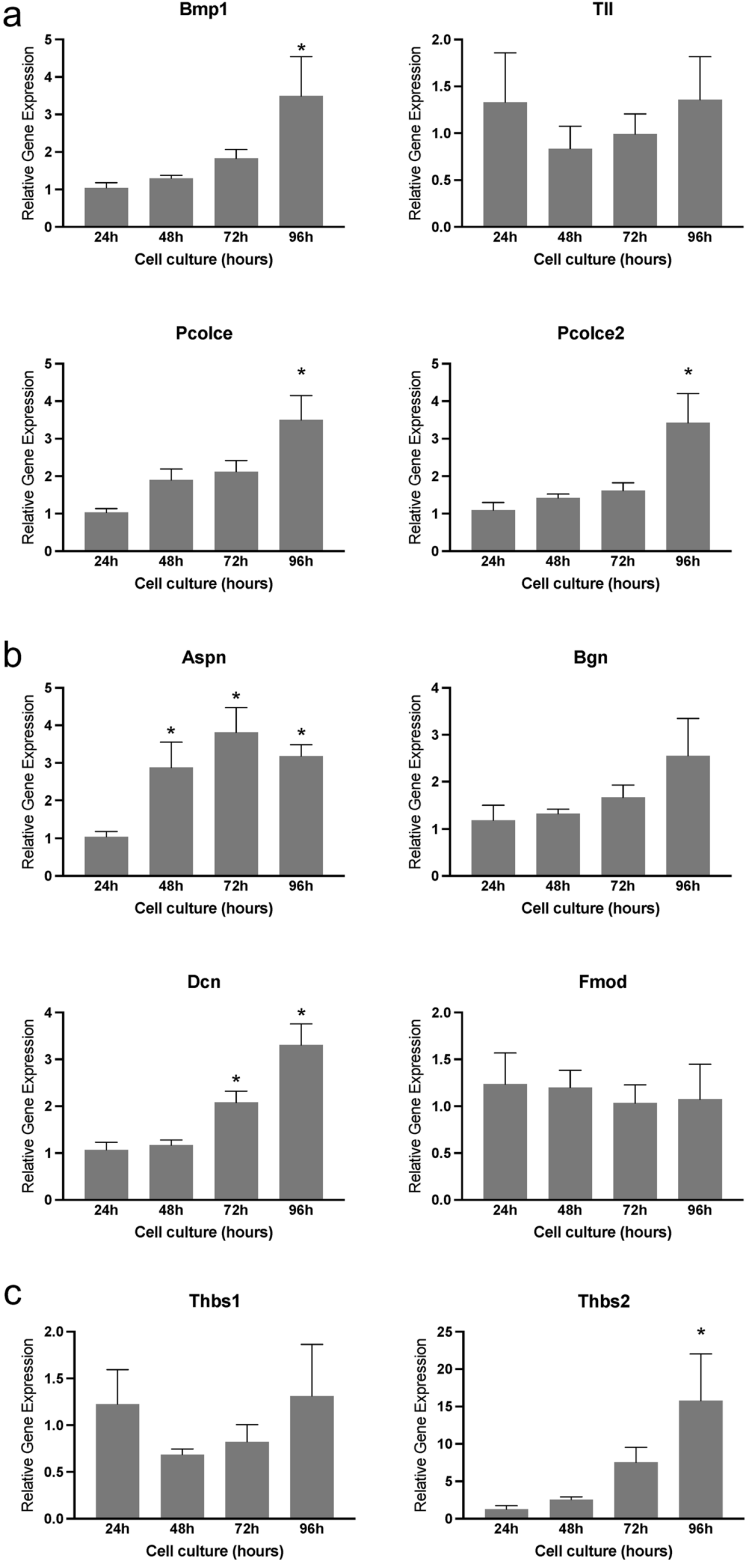
Gene expression of factors involved in the synthesis, processing, and assembly of fibrillar collagen in mouse endometrial stromal cells, decidualized in vitro. **a** Gene expression of Bmp1, Tll, Pcolce, and Pcolce2, in the mouse endometrial stromal cells decidualized in vitro. **b** Gene expression of genes encoding small leucine-rich proteoglycans such as Aspn, Bgn, Dcn, and Fmod in the mouse endometrial stromal cells decidualized in vitro. **c** Gene expression of Thbs1 and 2 in the mouse endometrial stromal cells decidualized in vitro. Mouse endometrial stromal cells isolated from gestational day 3 uteri were cultured in vitro. The cell lysates were collected at 24, 48, 72, and 96 h to extract total RNA for gene expression analysis. The gene expression was normalized to Rplp0 and compared with 24 h samples (*n* = 6/group, *p < 0.05)

To further understand the process of fibrillar collagen reorganization, we examined the expression of genes involved in its synthesis, processing, and assembly. Procollagen molecules, the soluble precursors of collagen, consist of globular pro-peptides at their N and C ends. The proteolytic action of procollagen N and C proteinases converts procollagen into mature collagen. These proteinases encompass bone morphogenetic protein 1 (BMP1), tolloid-like families (Tll), and a disintegrin and metalloproteinase with thrombospondin motif (ADAMTS). The activity of procollagen proteinases is regulated by enhancers, such as procollagen C-endopeptidase enhancer-1 (Pcolce) and procollagen C-endopeptidase enhancer-2 (Pcolce2) (Ricard-Blum 2011; Trackman 2005). Notably, the expression of Bmp1, Pcolce, and Pcolce2 gradually increases over time during stromal cell decidualization in vitro, reaching a peak significant level at 96 h, while Tll1 levels remain constant throughout (Fig. 3a).

Small leucine-rich proteoglycans (SLRPs), including asporin, biglycan, decorin, fibromodulin, and lumican, play a crucial role in collagen size, content, morphology, and growth rate during microfibril growth and merging into fibrils in both the longitudinal and axial directions (Kalamajski and Oldberg 2010; Chen and Birk 2013). The gene expression of these SLRPs reveals that asporin and decorin exhibit significant induction during decidualization compared to other factors (Fig. 3b). Thrombospondin-1 (Thbs1) and thrombospondin-2 (Thbs2) have both direct and indirect effects on collagen homeostasis (Bornstein et al. 2004; Rosini et al. 2018). Notably, the expression of Thbs2 is significantly elevated at 96 h, while Thbs1 levels remain unchanged (Fig. 3c). These findings suggest that the genes encoding factors influencing collagen synthesis, processing, and assembly exhibit differential regulation within the decidua, indicating alterations in the synthesis and processing of fibrillar collagen during endometrial decidualization.

### Elastic fiber reorganization within the mouse decidua during embryo implantation

**Fig. 4:**
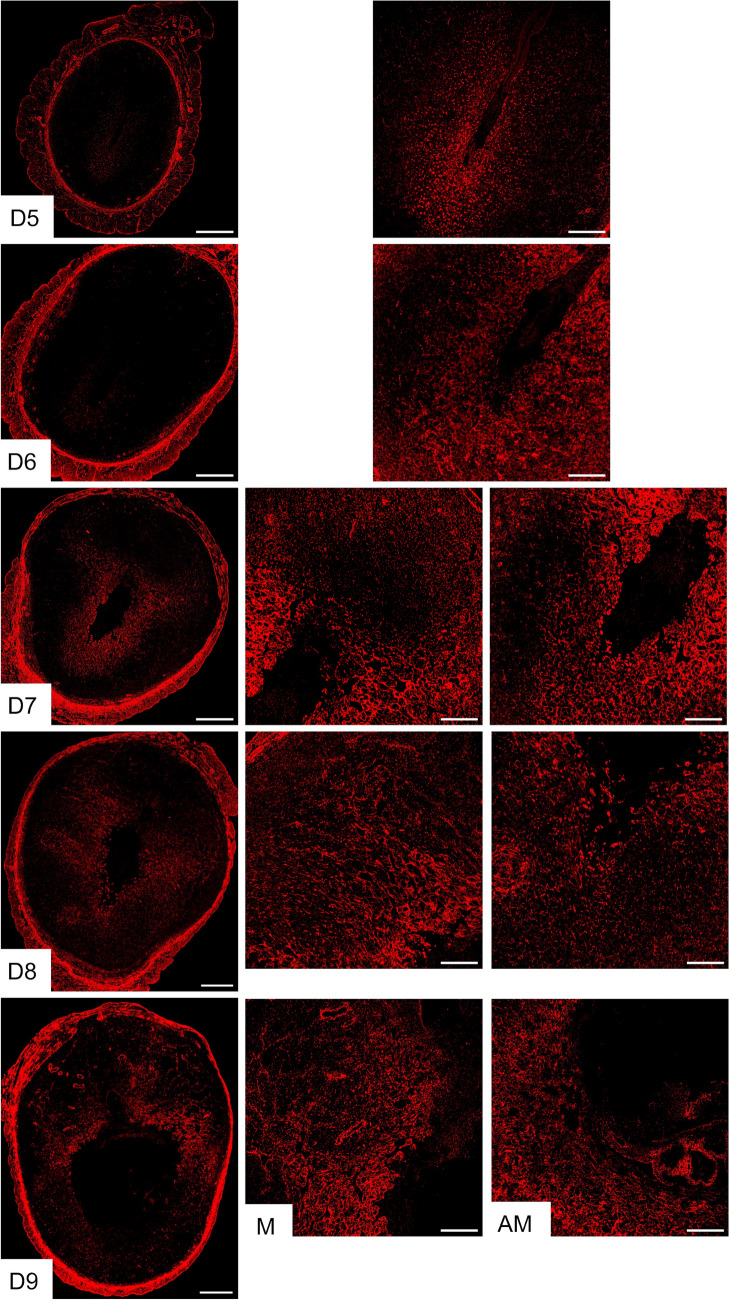
Localization of elastin in the mouse endometrium during early pregnancy. Confocal imaging of elastin in the frozen mouse uterine cross sections from gestational day 5 through 9. Left panel: Panoramic view of whole uterine sections; AM—Images captured from the anti-mesometrial region; M—Images captured from the mesometrial region. Representative images from three independent replicates. The imaging settings were adjusted for each individual image to optimize the visualization of morphology. Scale bar: 500 μm (left panel) and 200 μm (all other images)

Elastic fibers, in conjunction with collagen, constitute the fibrous components of the ECM and are an integral component of the vasculature, conferring tissues with elasticity and resilience (Wagenseil and Mecham 2007; Schmelzer and Duca 2021). These properties render elastic fibers not only indispensable for the integrity of the decidua but also essential for decidual angiogenesis. The decidua undergoes transformation into the maternal placenta during placentation, which is predominantly composed of an intricate network of vasculature (Turco and Moffett 2019). Therefore, we focused on the reorganization of elastic fibers within the decidua. Elastic fibers are composed of cross-linked tropoelastin embedded on microfibrils. Microfibrils are primarily made up of fibrillin and contain other proteins like EMILIN-1, fibulins, and microfibril-associated glycoproteins (Wagenseil and Mecham 2007). To visualize the reorganization of elastic fibers in the decidua, we used confocal imaging to detect elastin. Our observations showed that elastin expression began to appear around the implanted embryo within the decidua on gestational day 5 and gradually increased to cover the entire decidua by gestational day 8 (Fig. 4). Notably, on gestational day 9, the elastin was heavily localized in the blood vessels located within the mesometrial region, which will later form the placenta.

**Fig. 5:**
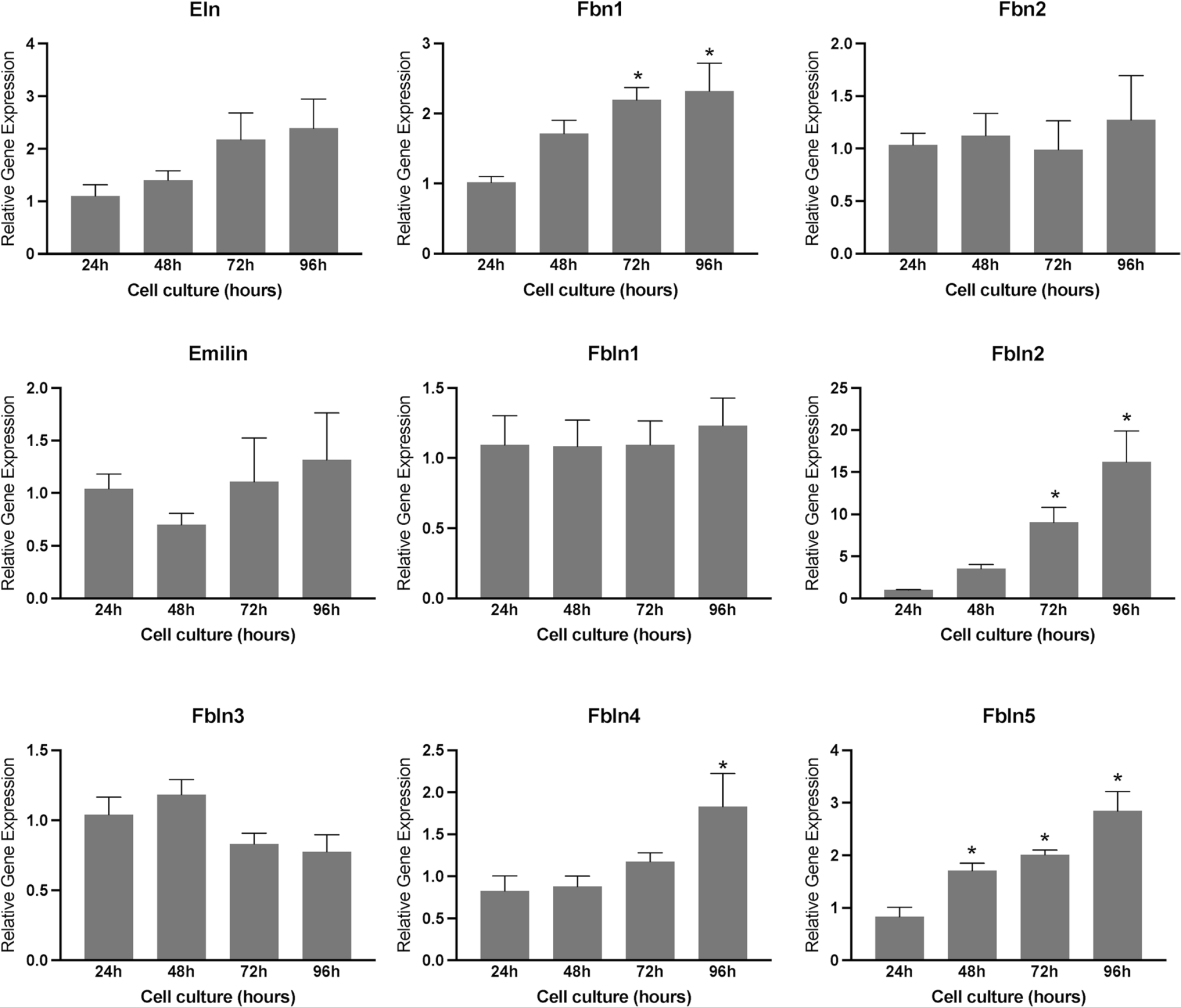
Gene expression of factors involved in the synthesis, processing, and assembly of elastic fiber in mouse endometrial stromal cells decidualized in vitro. Gene expression of Eln, Fbn1, Fbn2, Emillin, Fbln1, Fbln2, Fbln3, Fbln4, and Fbln5 in the mouse endometrial stromal cells decidualized in vitro. Mouse endometrial stromal cells isolated from gestational day 3 uteri were cultured in vitro. The cell lysates were collected at 24, 48, 72, and 96 h to extract total RNA for gene expression analysis. The gene expression was normalized to Rplp0 and compared with 24 h samples (*n* = 6/group, *p < 0.05)

Subsequently, we investigated the role of endometrial stromal cells in the production of elastic fibers. To achieve this objective, we conducted a comprehensive analysis of gene expression related to elastin synthesis, processing, and assembly in cell lysates obtained from endometrial stromal cell culture. While the expression of elastin, fibrillin 2, emilin, and fibulin 1 and 3 was detected, no significant difference was observed during endometrial decidualization in vitro. However, the genes such as fibrillin1 and fibulin 2, 4, and 5 were induced significantly (Fig. 5). These findings suggest that the reorganization of elastic fibers in the endometrium during decidualization may assume a pivotal role in the process of placentation.

### Expression and localization of lysyl oxidases in the decidua during embryo implantation

**Fig. 6:**
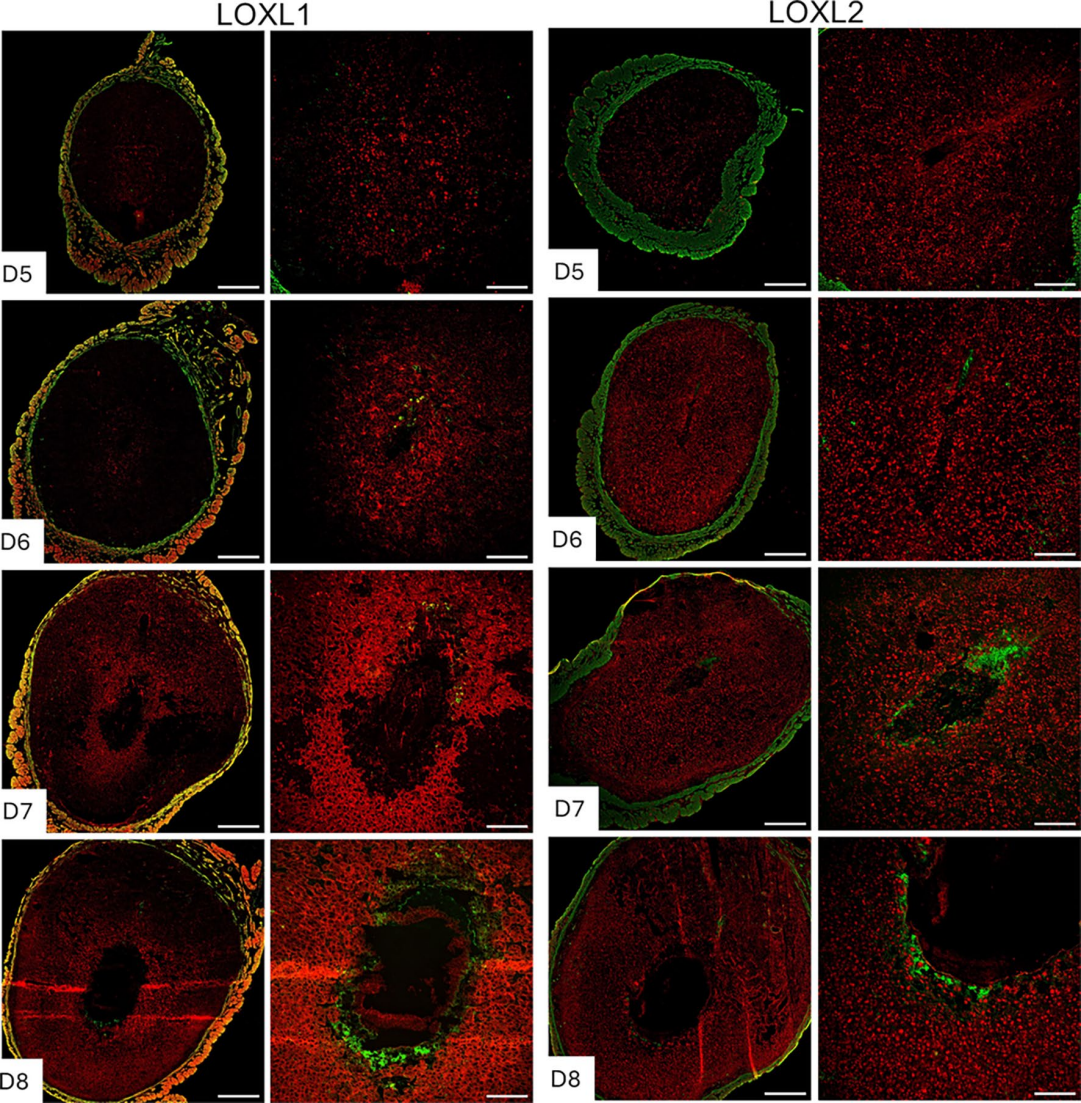
Localization of LOXL1 and LOXL2 in the mouse endometrium during embryo implantation. Confocal imaging of LOXL1 and LOXL2 in the frozen mouse uterine cross sections from gestational days 5 through 8. Left panels: Panoramic view of whole uterine sections. Red—LOXL1 or LOXL2; Green—Smooth muscle actin. Right panels: Images captured in decidua. Representative images from three independent replicates. The imaging settings were adjusted for each individual image to optimize the visualization of morphology. Scale bar: 500 μm (left panel) and 200 μm (all other images)

**Fig. 7:**
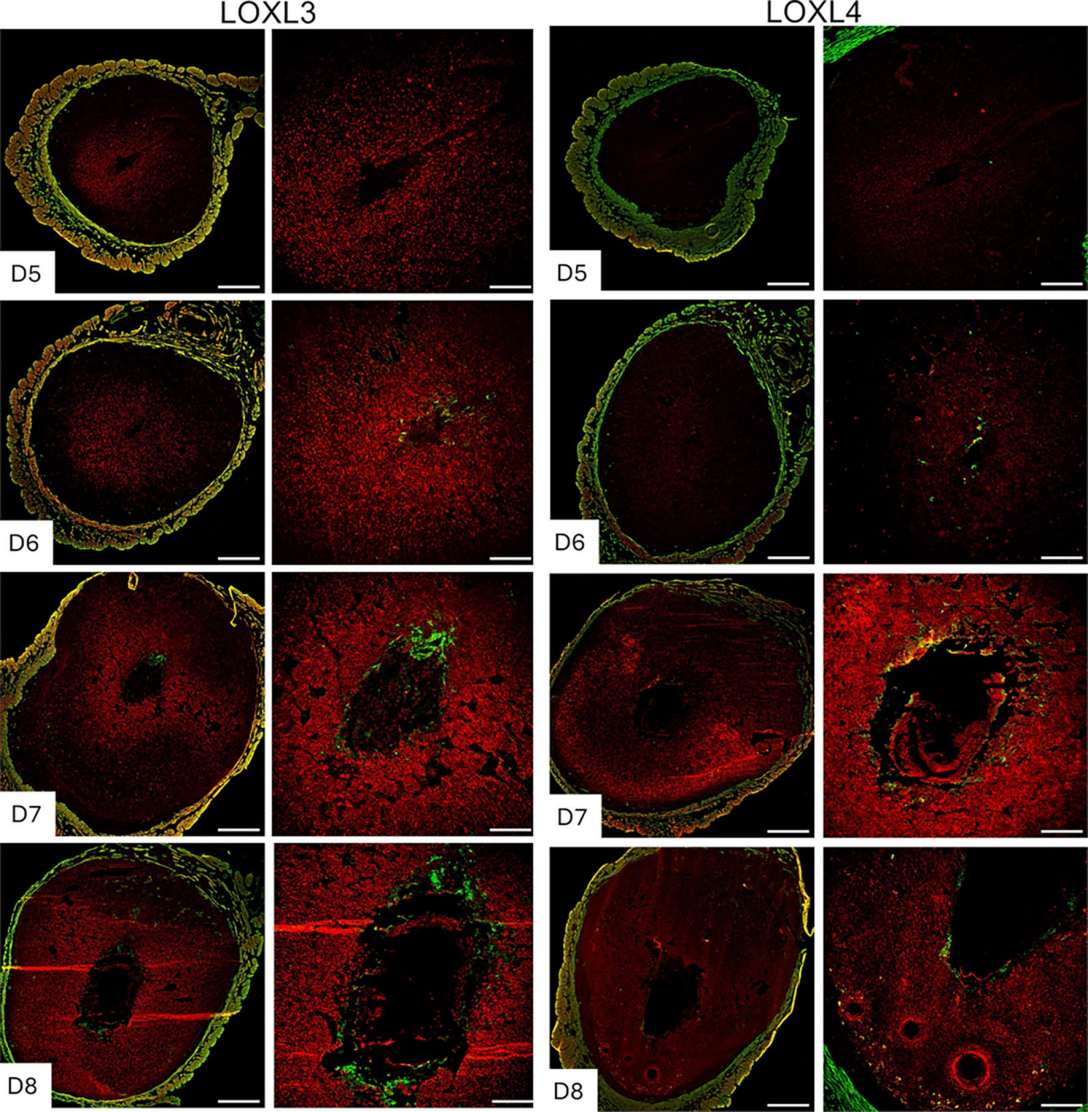
Localization of LOXL3 and LOXL4 in the mouse endometrium during embryo implantation. Confocal imaging of LOXL3 and LOXL4 in the frozen mouse uterine cross sections from gestational days 5 through 8. Left panels: Panoramic view of whole uterine sections. Red—LOXL3 or LOXL4; Green—Smooth muscle actin. Right panels: Images captured in decidua. Representative images from three independent replicates. The imaging settings were adjusted for each individual image to optimize the visualization of morphology. Scale bar: 500 μm (left panel) and 200 μm (all other images)

Collagen and elastic fibrous proteins are primarily strengthened by covalent cross-linking of lysine residues, catalyzed by the lysyl oxidase family of enzymes (Barker et al. 2012; Kagan and Li 2003). This family comprises five members: Lysyl oxidase (LOX) and LOX-like 1 to 4 (LOXL1-4). These enzymes share the uniform ability to cross-link collagen and elastic fibers, but they exhibit tissue-specific spatiotemporal expression and play multiple roles in the ECM and tissue function (Mäki 2009; Zaffryar-Eilot and Hasson 2022). Consequently, we investigated the expression of lysyl oxidases during decidualization. LOX has been reported to be expressed within the decidua during embryo implantation (Li et al. 2017). In this study, we focused on the localization of LOXL1-4 in the decidua. LOXL1 expression was modest within the decidua surrounding the implanted embryo, but it gradually expanded spatially from gestational days 7 to 8. LOXL2 was evenly distributed throughout the decidua from gestational days 5 to 8 (Fig. 6). LOXL3 was primarily localized in the primary decidual zone on gestational days 5 and 6, then gradually expanded throughout the decidua from gestational days 7 to 8. LOXL4 was first detected on gestational day 6 and diffusely expressed in the secondary decidual region on gestational day 7, then became widespread throughout the decidua on gestational day 8 (Fig. 7). These findings demonstrate that all lysyl oxidases are expressed in the endometrium during decidualization

**Fig. 8:**
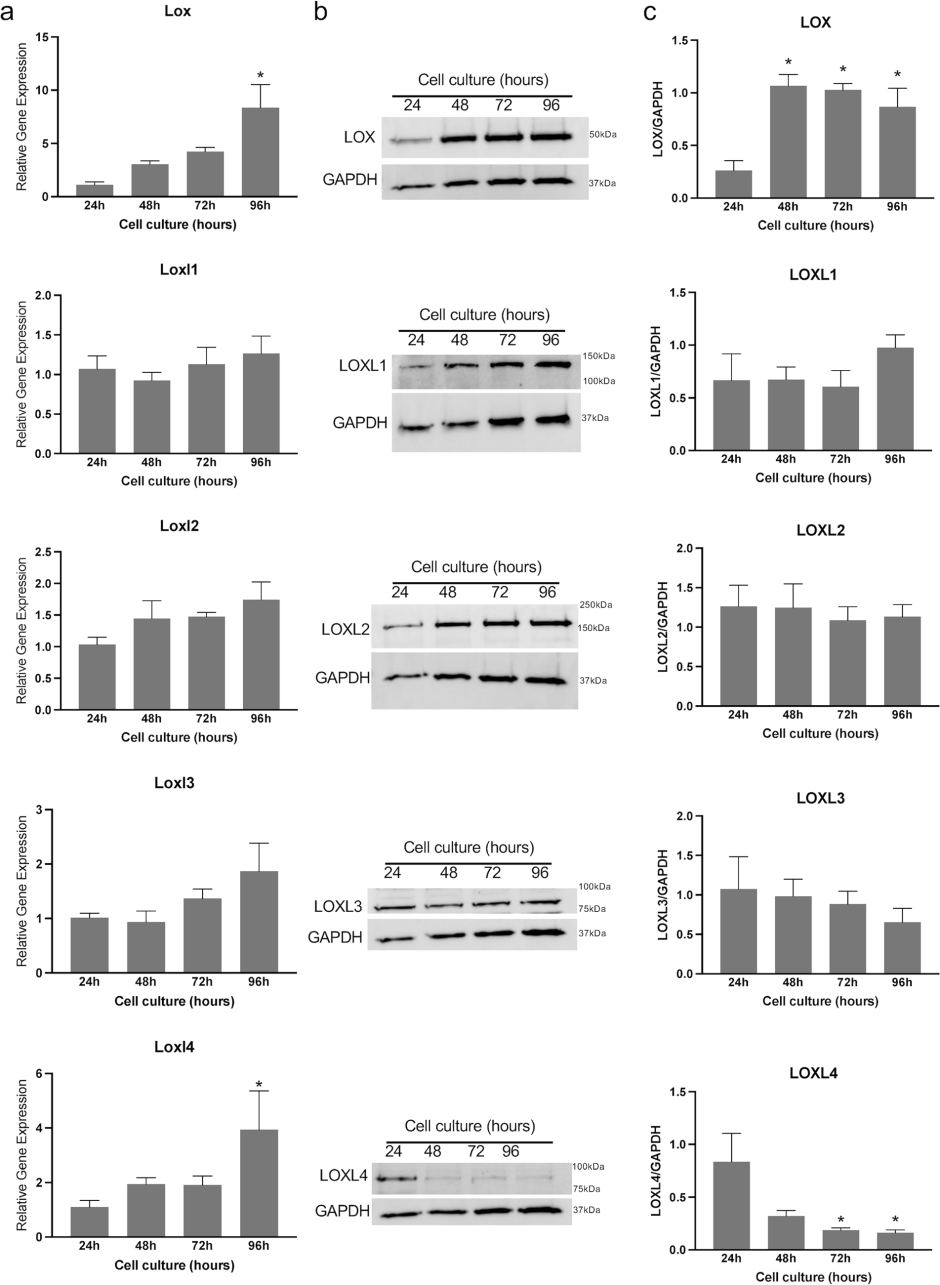
Gene expression and protein levels of LOX and LOXL1-4 in mouse endometrial stromal cells decidualized in vitro. **a** Gene expression of Lox and LOXL1-4 in the mouse endometrial stromal cells decidualized in vitro. Mouse endometrial stromal cells isolated from gestational day 3 uteri were cultured in vitro. The cell lysates were collected at 24, 48, 72, and 96 h to extract total RNA for gene expression analysis. The gene expression was normalized to Rplp0 and compared with 24 h samples (*n* = 6/group, *p < 0.05). **b** Western blot analysis of LOX and LOXL1-4 protein levels in cell lysates collected at 24, 48, 72, and 96 h. GAPDH was used as a loading control. These are representative images from three independent replicates. **c** Quantification of protein levels of LOX and LOXL1-4 is shown in histogram (*n* = 3/group, *p < 0.05)

To elucidate the role of endometrial stromal cells in the synthesis of lysyl oxidases, we analyzed the gene and protein expression of lysyl oxidases in lysates derived from endometrial stromal cell cultures. Our findings revealed the expression of all five lysyl oxidase genes in decidual cells. Among all genes, Lox and Loxl4 were significantly induced at 96 h (Fig. 8a). Consistent with gene expression, the protein level of LOX was significantly elevated from 48 to 96 h compared to 24 h. LOXL1, LOXL2, and LOXL3 levels remained unchanged throughout the in vitro decidualization period. In contrast to its gene expression, LOXL4 protein levels were significantly reduced during in vitro decidualization (Fig. 8b, c). This discrepancy could be attributed to its post-translational processing within the ECM. These findings suggest that lysyl oxidases are synthesized by endometrial stromal cells during decidualization and might play a crucial role in modifying the structural integrity of the decidua by stabilizing collagen and elastic fibers.

## Discussion

This study elucidates the ECM reorganization that occurs during endometrial decidualization, a pivotal phase in embryo implantation. It provides an in-depth analysis of the expression, distribution, and reorganization of fibrillar collagens, elastic fibers, and lysyl oxidases within the decidua during the implantation period. We have observed a rise in fibrillar collagen 1 levels and a decline in collagen 3 levels during the decidualization process. These findings are consistent with the reduction in the ratio of collagen type 3 to type 1 in human decidua (Iwahashi et al. 1996; Smith et al. 2016). Utilizing SHG, a highly sophisticated tool capable of mapping fibrillar collagen structure deeper into tissues, we have recorded the fibrillar collagen reorganization of the decidual tissues surrounding the invading embryo over the course of embryo implantation. The remarkable alterations in fiber structure and orientation between preimplantation and decidualized uterine stroma offer a novel perspective on the process of ECM reorganization during decidualization. The attachment of the embryo to the uterine epithelium and its subsequent invasion into the uterine stromal compartment occur in the mesometrium to anti-mesometrial orientation. This process remains an enigma for endometrial biologists. The collagen fiber orientation within the decidua during embryo invasion provides a potential clue to this process. Based on our observations obtained through SHG imaging of fibrillar collagen, we propose that the reorientation of the collagen fibers in parallel to the invading embryo may contribute to the direction of mesometrial to anti-mesometrial invasion of the embryo within the decidua.

Elastic fibers, a crucial fibrous component that collaborates with collagen to maintain the ECM architecture, are also an integral component of the vascular wall, providing tissue elasticity and resilience (Wagenseil and Mecham 2009, 2007). Elastin is highly localized in the mesometrial decidua (the destined site of placentation) and its blood vessels on gestational day 9. This spatial distribution of elastic fibers within the decidua over the implantation period suggests a potential role in decidual angiogenesis and, consequently, placentation. The strength and stability of collagen and elastic fibers are attributed to their cross-linking mediated by lysyl oxidases (Zaffryar-Eilot and Hasson 2022; Barker et al. 2012). Although these lysyl oxidases are widely expressed in collagen and elastin-containing tissues, their spatiotemporal expression pattern varies across different tissues (Zaffryar-Eilot and Hasson 2022). The robust spatial distribution of these lysyl oxidases within the decidua during embryo implantation implicates their potential role in embryo implantation.

The forceful invasion of trophoblast into decidual tissues has the potential to alter the structural integrity and, consequently, the mechanical properties of the decidua. Therefore, the decidua should possess appropriate mechanisms to regulate trophoblast invasion without compromising its tissue mechanical homeostasis. The embryo invasion and its impact on decidual tissue stiffness throughout decidualization remain poorly understood. Based on our current understanding, we propose that ECM reorganization during decidualization assumes this responsibility to maintain decidual tissue mechanical homeostasis by precisely regulating embryo invasion. This hypothesis is supported by clinical complications such as shallow implantation and placenta accreta spectrum disorders. Unregulated, deeper trophoblast invasion that penetrates the myometrium can lead to placenta accreta, while inadequate invasion results in shallow implantation (Stanek 2023; O’Connor et al. 2020; Norwitz 2006). The rapid proliferation and differentiation of stromal cells lead to the development of enlarged tissue growth at the implantation sites, known as decidua or deciduoma. Although not a true neoplasm, decidua exhibits tissue growth and ECM reorganization, similar to neoplastic processes. Notably, fibrillar collagen reorganization is closely associated with tumor tissue stiffness in neoplasms (Cox 2021; Han et al. 2016; Levental et al. 2009; Kai et al. 2019). Aberrant collagen reorganization and elevated lysyl oxidase levels have also been observed in endometrial pathologies, such as endometriosis (Kim et al. 2019; Ruiz et al. 2015). Consequently, it is imperative to expand our understanding of endometrial tissue mechanical properties during embryo implantation.

ECM is predominantly synthesized by stromal cells, although most other cell types, such as smooth muscle cells, immune cells, and vascular endothelial cells, are also known to synthesize ECM constituents (DeLeon-Pennell et al. 2020; Wagenseil and Mecham 2009). Utilizing purification and culture of endometrial stromal cells, we have unequivocally demonstrated that uterine stromal cells can synthesize fibrillar collagen, elastin, and lysyl oxidases during decidualization even in the absence of embryo invasion. These findings confirm that endometrial stromal cells assume a central role in orchestrating this ECM reorganization. Embryonic life customizes ECM, which undergoes limited reorganization in adulthood, except in reproductive tissues (Bonnans et al. 2014; Nallasamy et al. 2017; Liu et al. 2004). ECM reorganization, including its rapid degradation and turnover, has been reported in various reproductive tissues (Nallasamy et al. 2021; Morrione and Seifter 1962; Woessner and Brewer 1963). The timely ECM reorganization is crucial for reproductive tissues to fulfill their functions, which are contingent on the dynamic reproductive statuses of non-pregnancy and pregnancy. For instance, the mechanisms involved in ECM reorganization in the myometrium and cervix to perform opposing functions during pregnancy and parturition have been elucidated (Nallasamy et al. 2018, 2021, 2017; Ouellette et al. 2022). Any aberrant ECM reorganization in these tissues can lead to parturition defects such as dystocia and preterm birth (Nallasamy et al. 2018; Ouellette et al. 2025). Defective ECM, particularly elastic fibers and their constituents, in the pelvic floor is the predominant cause of pelvic organ prolapse (Drewes et al. 2007; Liu et al. 2004). These studies underscore the significance of ECM reorganization in the reproductive tract. Our knowledge regarding ECM reorganization in the endometrium is still limited to the processes of decidualization, placentation, and endometrial regeneration.

The present study elucidates several clues involved in ECM reorganization during endometrial decidualization and embryo implantation. Future research endeavors within our laboratory will involve the generation of mouse models deficient in specific ECM components to ascertain their functional relevance in vivo during these processes.

## Supplementary Information

Below is the link to the electronic supplementary material.

Supplementary file1 (DOCX 3364 KB)

## Data Availability

No datasets were generated or analyzed during the current study.

## References

[CR1] Akaeda S, Aikawa S, Hirota Y (2024) Spatial and molecular anatomy of the endometrium during embryo implantation: a current overview of key regulators of blastocyst invasion. FEBS J 291(19):4206–4221. 10.1111/febs.17077 38348632 10.1111/febs.17077

[CR2] Barker HE, Cox TR, Erler JT (2012) The rationale for targeting the LOX family in cancer. Nat Rev Cancer 12(8):540–552. 10.1038/nrc3319 22810810 10.1038/nrc3319

[CR3] Bonnans C, Chou J, Werb Z (2014) Remodelling the extracellular matrix in development and disease. Nat Rev Mol Cell Biol 15(12):786–801. 10.1038/nrm3904 25415508 10.1038/nrm3904 PMC4316204

[CR4] Bornstein P, Agah A, Kyriakides TR (2004) The role of thrombospondins 1 and 2 in the regulation of cell–matrix interactions, collagen fibril formation, and the response to injury. Int J Biochem Cell Biol 36(6):1115–1125. 10.1016/j.biocel.2004.01.012 15094126 10.1016/j.biocel.2004.01.012

[CR5] Campagnola P (2011) Second harmonic generation imaging microscopy: applications to diseases diagnostics. Anal Chem 83(9):3224–3231. 10.1021/ac1032325 21446646 10.1021/ac1032325 PMC3104727

[CR6] Cha J, Sun X, Dey SK (2012) Mechanisms of implantation: strategies for successful pregnancy. Nat Med 18(12):1754–1767. 10.1038/nm.3012 23223073 10.1038/nm.3012 PMC6322836

[CR7] Chaudhuri O, Cooper-White J, Janmey PA, Mooney DJ, Shenoy VB (2020) Effects of extracellular matrix viscoelasticity on cellular behaviour. Nature 584(7822):535–546. 10.1038/s41586-020-2612-2 32848221 10.1038/s41586-020-2612-2 PMC7676152

[CR8] Chen S, Birk DE (2013) The regulatory roles of small leucine-rich proteoglycans in extracellular matrix assembly. FEBS J 280(10):2120–2137. 10.1111/febs.12136 23331954 10.1111/febs.12136 PMC3651807

[CR9] Cheong M-L, Lai T-H, Wu W-B (2019) Connective tissue growth factor mediates transforming growth factor β-induced collagen expression in human endometrial stromal cells. PLoS ONE 14(1):e0210765. 10.1371/journal.pone.0210765 30695033 10.1371/journal.pone.0210765 PMC6350958

[CR10] Cox TR (2021) The matrix in cancer. Nat Rev Cancer 21(4):217–238. 10.1038/s41568-020-00329-7 33589810 10.1038/s41568-020-00329-7

[CR11] DeLeon-Pennell KY, Barker TH, Lindsey ML (2020) Fibroblasts: the arbiters of extracellular matrix remodeling. Matrix Biol 91–92:1–7. 10.1016/j.matbio.2020.05.006 32504772 10.1016/j.matbio.2020.05.006 PMC7434687

[CR12] Drewes PG, Yanagisawa H, Starcher B, Hornstra I, Csiszar K, Marinis SI, Keller P, Word RA (2007) Pelvic organ prolapse in fibulin-5 knockout mice: pregnancy-induced changes in elastic fiber homeostasis in mouse vagina. Am J Pathol 170(2):578–589. 10.2353/ajpath.2007.060662 17255326 10.2353/ajpath.2007.060662 PMC1851882

[CR13] Favaro R, Abrahamsohn PA, Zorn MT (2014) Decidualization and endometrial extracellular matrix remodeling. In: Croy BA, Yamada AT, DeMayo FJ, Adamson SL (eds) The guide to investigation of mouse pregnancy. Academic Press, Boston, pp 125–142

[CR14] Gellersen B, Brosens JJ (2014) Cyclic decidualization of the human endometrium in reproductive health and failure. Endocr Rev 35(6):851–905. 10.1210/er.2014-1045 25141152 10.1210/er.2014-1045

[CR15] Han W, Chen S, Yuan W, Fan Q, Tian J, Wang X, Chen L, Zhang X, Wei W, Liu R, Qu J, Jiao Y, Austin RH, Liu L (2016) Oriented collagen fibers direct tumor cell intravasation. Proc Natl Acad Sci U S A 113(40):11208–11213. 10.1073/pnas.1610347113 27663743 10.1073/pnas.1610347113 PMC5056065

[CR16] Herndon CN, Aghajanova L, Balayan S, Erikson D, Barragan F, Goldfien G, Vo KC, Hawkins S, Giudice LC (2016) Global transcriptome abnormalities of the eutopic endometrium from women with adenomyosis. Reprod Sci 23(10):1289–1303. 10.1177/1933719116650758 27233751 10.1177/1933719116650758 PMC6344825

[CR17] Humphrey JD, Dufresne ER, Schwartz MA (2014) Mechanotransduction and extracellular matrix homeostasis. Nat Rev Mol Cell Biol 15(12):802–812. 10.1038/nrm3896 25355505 10.1038/nrm3896 PMC4513363

[CR18] Iwahashi M, Muragaki Y, Ooshima A, Yamoto M, Nakano R (1996) Alterations in distribution and composition of the extracellular matrix during decidualization of the human endometrium. J Reprod Fertil 108(1):147–155. 10.1530/jrf.0.1080147 8958841 10.1530/jrf.0.1080147

[CR19] Kagan HM, Li W (2003) Lysyl oxidase: properties, specificity, and biological roles inside and outside of the cell. J Cell Biochem 88(4):660–672. 10.1002/jcb.10413 12577300 10.1002/jcb.10413

[CR20] Kai F, Drain AP, Weaver VM (2019) The extracellular matrix modulates the metastatic journey. Dev Cell 49(3):332–346. 10.1016/j.devcel.2019.03.026 31063753 10.1016/j.devcel.2019.03.026 PMC6527347

[CR21] Kalamajski S, Oldberg A (2010) The role of small leucine-rich proteoglycans in collagen fibrillogenesis. Matrix Biol 29(4):248–253. 10.1016/j.matbio.2010.01.001 20080181 10.1016/j.matbio.2010.01.001

[CR22] Kim TH, Yoo JY, Choi KC, Shin JH, Leach RE, Fazleabas AT, Young SL, Lessey BA, Yoon HG, Jeong JW (2019) Loss of HDAC3 results in nonreceptive endometrium and female infertility. Sci Transl Med. 10.1126/scitranslmed.aaf7533 30626716 10.1126/scitranslmed.aaf7533 PMC6650287

[CR23] Levental KR, Yu H, Kass L, Lakins JN, Egeblad M, Erler JT, Fong SFT, Csiszar K, Giaccia A, Weninger W, Yamauchi M, Gasser DL, Weaver VM (2009) Matrix crosslinking forces tumor progression by enhancing integrin signaling. Cell 139(5):891–906. 10.1016/j.cell.2009.10.027 19931152 10.1016/j.cell.2009.10.027 PMC2788004

[CR24] Li S-Y, Yan J-q, Song Z, Liu Y-F, Song M-J, Qin J-W, Yang Z-M, Liang X-H (2017) Molecular characterization of lysyl oxidase-mediated extracellular matrix remodeling during mouse decidualization. FEBS Lett 591(10):1394–1407. 10.1002/1873-3468.12645 28380254 10.1002/1873-3468.12645

[CR25] Liu X, Zhao Y, Gao J, Pawlyk B, Starcher B, Spencer JA, Yanagisawa H, Zuo J, Li T (2004) Elastic fiber homeostasis requires lysyl oxidase-like 1 protein. Nat Genet 36(2):178–182. 10.1038/ng1297 14745449 10.1038/ng1297

[CR26] Lu P, Takai K, Weaver VM, Werb Z (2011) Extracellular matrix degradation and remodeling in development and disease. Cold Spring Harb Perspect Biol. 10.1101/cshperspect.a005058 21917992 10.1101/cshperspect.a005058 PMC3225943

[CR27] Mäki JM (2009) Lysyl oxidases in mammalian development and certain pathological conditions. Histol Histopathol 24(5):651–660. 10.14670/hh-24.651 19283672 10.14670/HH-24.651

[CR28] Morrione TG, Seifter S (1962) Alteration in the collagen content of the human uterus. J Exp Med 115(2):357–365 14476228 10.1084/jem.115.2.357 PMC2137490

[CR29] Mouw JK, Ou G, Weaver VM (2014) Extracellular matrix assembly: a multiscale deconstruction. Nat Rev Mol Cell Biol 15(12):771–785. 10.1038/nrm3902 25370693 10.1038/nrm3902 PMC4682873

[CR30] Muter J, Lynch VJ, McCoy RC, Brosens JJ (2023) Human embryo implantation. Development. 10.1242/dev.201507 37254877 10.1242/dev.201507 PMC10281521

[CR31] Naba A (2024) Mechanisms of assembly and remodelling of the extracellular matrix. Nat Rev Mol Cell Biol 25(11):865–885. 10.1038/s41580-024-00767-3 39223427 10.1038/s41580-024-00767-3 PMC11931590

[CR32] Nallasamy S, Yoshida K, Akins M, Myers K, Iozzo R, Mahendroo M (2017) Steroid hormones are key modulators of tissue mechanical function via regulation of collagen and elastic fibers. Endocrinology 158(4):950–962. 10.1210/en.2016-1930 28204185 10.1210/en.2016-1930 PMC5460796

[CR33] Nallasamy S, Akins M, Tetreault B, Luby-Phelps K, Mahendroo M (2018) Distinct reorganization of collagen architecture in lipopolysaccharide-mediated premature cervical remodeling. Biol Reprod 98(1):63–74. 10.1093/biolre/iox155 29161343 10.1093/biolre/iox155 PMC5803761

[CR34] Nallasamy S, Palacios HH, Setlem R, Caraballo MC, Li K, Cao E, Shankaran M, Hellerstein M, Mahendroo M (2021) Transcriptome and proteome dynamics of cervical remodeling in the mouse during pregnancy. Biol Reprod. 10.1093/biolre/ioab144 34309663 10.1093/biolre/ioab144 PMC8599062

[CR35] Norwitz ER (2006) Defective implantation and placentation: laying the blueprint for pregnancy complications. Reprod Biomed Online 13(4):591–599. 10.1016/s1472-6483(10)60649-9 17007686 10.1016/s1472-6483(10)60649-9

[CR36] O’Connor BB, Pope BD, Peters MM, Ris-Stalpers C, Parker KK (2020) The role of extracellular matrix in normal and pathological pregnancy: Future applications of microphysiological systems in reproductive medicine. Exp Biol Med (Maywood) 245(13):1163–1174. 10.1177/1535370220938741 32640894 10.1177/1535370220938741 PMC7400725

[CR37] Ochoa-Bernal MA, Fazleabas AT (2020) Physiologic events of embryo implantation and decidualization in human and non-human primates. Int J Mol Sci. 10.3390/ijms21061973 32183093 10.3390/ijms21061973 PMC7139778

[CR38] Ouellette A, Mahendroo M, Nallasamy S (2022) Collagen and elastic fiber remodeling in the pregnant mouse myometrium. Biol Reprod. 10.1093/biolre/ioac102 35594450 10.1093/biolre/ioac102 PMC9767674

[CR39] Ouellette A, Do C, Cohn-Guthrie S, Lam YW, Mahendroo M, Nallasamy S (2025) Lysyl oxidases are necessary for myometrial contractility and on-time parturition in mice. J Endocr Soc 9(5):bvaf028. 10.1210/jendso/bvaf028 40170697 10.1210/jendso/bvaf028 PMC11959360

[CR40] Pathare ADS, Loid M, Saare M, Gidlöf SB, Zamani Esteki M, Acharya G, Peters M, Salumets A (2023) Endometrial receptivity in women of advanced age: an underrated factor in infertility. Hum Reprod Update 29(6):773–793. 10.1093/humupd/dmad019 37468438 10.1093/humupd/dmad019 PMC10628506

[CR41] Ramathal CY, Bagchi IC, Taylor RN, Bagchi MK (2010) Endometrial decidualization: of mice and men. Semin Reprod Med 28(1):17–26. 10.1055/s-0029-1242989 20104425 10.1055/s-0029-1242989 PMC3095443

[CR42] Ricard-Blum S (2011) The collagen family. Cold Spring Harb Perspect Biol 3(1):a004978–a004978. 10.1101/cshperspect.a004978 21421911 10.1101/cshperspect.a004978 PMC3003457

[CR43] Rosini S, Pugh N, Bonna AM, Hulmes DJS, Farndale RW, Adams JC (2018) Thrombospondin-1 promotes matrix homeostasis by interacting with collagen and lysyl oxidase precursors and collagen cross-linking sites. Sci Signal 11(532):eaar2566. 10.1126/scisignal.aar2566 29844053 10.1126/scisignal.aar2566

[CR44] Rossi F, Luppi S, Fejza A, Giolo E, Ricci G, Andreuzzi E (2025) Extracellular matrix and pregnancy: functions and opportunities caught in the net. Reprod Biol Endocrinol 23(1):24. 10.1186/s12958-025-01348-5 39953593 10.1186/s12958-025-01348-5 PMC11827249

[CR45] Ruiz LA, Baez-Vega PM, Ruiz A, Peterse DP, Monteiro JB, Bracero N, Beauchamp P, Fazleabas AT, Flores I (2015) Dysregulation of lysyl oxidase expression in lesions and endometrium of women with endometriosis. Reprod Sci 22(12):1496–1508. 10.1177/1933719115585144 25963914 10.1177/1933719115585144 PMC5933196

[CR46] Schmelzer CEH, Duca L (2021) Elastic fibers: formation, function, and fate during aging and disease. FEBS J. 10.1111/febs.15899 33896108 10.1111/febs.15899

[CR47] Shi JW, Lai ZZ, Yang HL, Yang SL, Wang CJ, Ao D, Ruan LY, Shen HH, Zhou WJ, Mei J, Fu Q, Li MQ (2020) Collagen at the maternal-fetal interface in human pregnancy. Int J Biol Sci 16(12):2220–2234. 10.7150/ijbs.45586 32549767 10.7150/ijbs.45586 PMC7294936

[CR48] Smith SD, Choudhury RH, Matos P, Horn JA, Lye SJ, Dunk CE, Aplin JD, Jones RL, Harris LK (2016) Changes in vascular extracellular matrix composition during decidual spiral arteriole remodeling in early human pregnancy. Histol Histopathol 31(5):557–571. 10.14670/hh-11-696 26602431 10.14670/HH-11-696

[CR49] Spiess K, Teodoro WR, Zorn TM (2007) Distribution of collagen types I, III, and V in pregnant mouse endometrium. Connect Tissue Res 48(2):99–108. 10.1080/03008200601166194 17453912 10.1080/03008200601166194

[CR50] Stanek J (2023) Shallow placentation: a distinct category of placental lesions. Am J Perinatol 40(12):1328–1335. 10.1055/s-0041-1735554 34587634 10.1055/s-0041-1735554

[CR51] Theocharis AD, Skandalis SS, Gialeli C, Karamanos NK (2016) Extracellular matrix structure. Adv Drug Deliv Rev 97:4–27. 10.1016/j.addr.2015.11.001 26562801 10.1016/j.addr.2015.11.001

[CR52] Trackman PC (2005) Diverse biological functions of extracellular collagen processing enzymes. J Cell Biochem 96(5):927–937. 10.1002/jcb.20605 16167328 10.1002/jcb.20605 PMC1352157

[CR53] Turco MY, Moffett A (2019) Development of the human placenta. Development. 10.1242/dev.163428 31776138 10.1242/dev.163428

[CR54] Vinketova K, Mourdjeva M, Oreshkova T (2016) Human decidual stromal cells as a component of the implantation niche and a modulator of maternal immunity. J Pregnancy 2016:8689436. 10.1155/2016/8689436 27239344 10.1155/2016/8689436 PMC4864559

[CR55] Wagenseil JE, Mecham RP (2007) New insights into elastic fiber assembly. Birth Defects Res C Embryo Today 81(4):229–240. 10.1002/bdrc.20111 18228265 10.1002/bdrc.20111

[CR56] Wagenseil JE, Mecham RP (2009) Vascular extracellular matrix and arterial mechanics. Physiol Rev 89(3):957–989. 10.1152/physrev.00041.2008 19584318 10.1152/physrev.00041.2008 PMC2775470

[CR57] WoessnerJun J, Brewer T (1963) Formation and breakdown of collagen and elastin in the human uterus pregnancy and post-partum involution. Biochem J 89(1):75–82. 10.1042/bj0890075 14097370 10.1042/bj0890075 PMC1202274

[CR58] Zaffryar-Eilot S, Hasson P (2022) Lysyl oxidases: orchestrators of cellular behavior and ecm remodeling and homeostasis. Int J Mol Sci. 10.3390/ijms231911378 36232685 10.3390/ijms231911378 PMC9569843

