# Supplementary material for: Extracellular matrix reorganization during endometrial decidualization
Source: Histochem Cell Biol. 2025 Aug 28;163(1):84. doi: 10.1007/s00418-025-02411-3 (PMC12394361; doi:10.1007/s00418-025-02411-3)
Supplement: Supplementary file 1 — Supplementary file1 (DOCX 3364 KB) [file 418_2025_2411_MOESM1_ESM.docx]

**Extracellular Matrix Reorganization During Endometrial Decidualization**

Mona Gebril^1^, Sparhawk Mulder^1^, Rimi Das^1^ and Shanmugasundaram Nallasamy^1,2^

^1^Department of Obstetrics, Gynecology, and Reproductive Sciences,

University of Vermont College of Medicine, Burlington, Vermont, USA

**^2^Corresponding author:** Shanmugasundaram Nallasamy, DVM, PhD

Assistant Professor

Division of Reproductive Sciences
Dept. of Obstetrics, Gynecology and Reproductive Sciences

University of Vermont College of Medicine

89 Beaumont Avenue, Burlington, VT 05405

E-mail: [Shanmugasundaram.Nallasamy@med.uvm.edu](mailto:Shanmugasundaram.Nallasamy@med.uvm.edu)

ORCID: 0000-0002-3161-6241

**Journal Name:** Histochemistry and Cell Biology

**Supplementary fig. 1 Localization of collagen 1 in the mouse endometrium during embryo implantation** Confocal imaging of COL1A1 in the frozen mouse uterine cross sections from gestation day 5 through 9. Left panel: Panoramic view of whole uterine sections; AM – Images captured from anti-mesometrial region; M - Images captured from mesometrial region. Representative images from three independent replicates. Scale bar: 500μm (left panel) and 200μm (all other images).

**Supplementary fig. 2 Localization of collagen 3 in the mouse endometrium during embryo implantation** Confocal imaging of collagen 3 in the frozen mouse uterine cross sections from gestation day 3, and 5 through 9. Left panel: Panoramic view of whole uterine sections; AM – Images captured from anti-mesometrial region; M - Images captured from mesometrial region. Representative images from three independent replicates. Scale bar: 500μm (left panel) and 200μm (all other images).
